# Evaluation via simulation of statistical corrections for network nonindependence

**DOI:** 10.1007/s10742-023-00311-4

**Published:** 2023-08-12

**Authors:** Luke J. Matthews, Megan S. Schuler, Raffaele Vardavas, Joshua Breslau, Ioana Popescu

**Affiliations:** 1 RAND Corporation, 20 Park Plaza #920, Boston, MA 02216, USA; 2 RAND Corporation, 1200 S Hayes Street, Arlington, VA 2220, USA; 3 RAND Corporation, 1776 Main St, Santa Monica, CA 90401, USA; 4 RAND Corporation, 4570 Fifth Ave #600, Pittsburgh, PA 15213, USA

**Keywords:** Network, Influence, Autocorrelation, Autoregression, Nonindependence, Sampling

## Abstract

Social processes and social context are increasingly recognized as key factors shaping health-related behaviors and outcomes. One social process that may be acting within social networks is social influence, in which an individual’s characteristic (e.g., specific health behavior) is potentially impacted by the corresponding characteristic of connected individuals in the network. In the health services context, healthcare providers who work together and share patients may influence each other through the knowledge transmission or development of clinical practice norms. Although many statistical techniques assume independence of data points, when analyzing data that may reflect social processes acting across a social network, it is imperative to account for the interdependencies (i.e., non-independence) across individuals. In practice, studies account for nonindependence in the context of estimating bivariate relations (correlations or linear regression) using a variety of analytic methods (some of which have previously been shown to yield biased results). To date, it is unclear which methods yield acceptable false positive rates, unbiased coefficient estimates, and acceptable statistical power, because there have been no systematic simulation studies comparing methods for addressing network nonindependence arising from social influence. To address this gap, we compared eight commonly used methods that purport to account for nonindependence using simulated network data. While results indicated that none of the techniques reduced false positive rates to the predicted (nominal) 0.05 level, random sampling of network nodes was the method that yielded the smallest false positive rates, yet came at a price of reduced statistical power. Further methodological development is needed.

## 1 Introduction

Social network analysis seeks to characterize the interrelationships between individuals (or other social entities) and can serve both to identify the network structure linking individuals as well as to examine how particular behaviors or health outcomes may be impacted by social ties. Social network analyses, long a staple in the fields of anthropology and sociology, are becoming more popular among health service researchers, given growing appreciation of the role that social context plays in shaping health-related behaviors and outcomes (O’Malley and Marsden 2008). Key mechanisms through which social relationships are hypothesized to impact health include: providing direct support and resources (including tangible resources, emotional support, information sharing); and shaping behavioral norms (Berkman and Glass 2000). In the health services context, one area of research interest is examining how social network connections between healthcare providers impact provider behavior and health outcomes. Specifically, it is hypothesized that providers who work together and share patients may influence each other through the transmission of information or development of mutually recognized norms of practice (Brunson and Laubenbacher 2018; Barnett et al. 2011; Breslau et al. 2021; Stein et al. 2017; Pollack et al. 2012a; Manchanda et al. 2008). For instance, if one provider adopts non-opioid treatment alternatives for pain disorders, closely linked colleagues may similarly alter their clinical practice. Other recent applications of social network analysis in the health context include: assessing the impact of provider team structure on health outcome and cost among Medicare patients (Kuo et al. 2020), examining the relationship between social network characteristics and living donor kidney transplantation (Gillespie et al. 2020), examining the impact of peer and family social network structure on adolescent drinking (McCann et al. 2019), and characterizing social transmission of positive and negative sentiment towards COVID-19 responses (Hung et al. 2020).

Unlike in many contexts where the analytic sample comprises individuals who are assumed to be an independent sample of the population, individuals constituting a social network do not reflect an independent sample. Indeed, the statistical independence of observed data points is a foundational assumption of most statistical techniques, including regression, comparisons of means, difference-in-differences approaches, instrumental variables, and many more (Naroll 1961a; Grafen and Hamilton 1989; Felsenstein 1985; Rohlf 2006). However, network data have long been known to violate the independence assumption because each data point (i.e., each “node,” often an individual) has a relational connection to other nodes within the social network structure. If a given observed characteristic (i.e., trait) of one node is potentially impacted by the corresponding characteristic of connected nodes in the network—e.g., through the process of social influence—then nodes cannot be assumed to be statistically independent (Leenders 2002). Rather, one individual’s trait value will directly impact the trait values of connected individuals via the social influence process. For example, while physician opioid prescribing behaviors and statin prescribing behaviors may be assumed to be independent, they both may be affected by information diffusion and social influence processes within physician social networks (O’Malley and Marsden 2008; Leenders 2002).

We note that a different problem of statistical nonindependence occurs when the traits of nodes induce connections to form between them, creating a higher likelihood of social ties between similar individuals (referred to as homophily). Although homophily and influence are not fully distinguishable in empirical data, they are conceptually distinct (Shalizi and Thomas 2011). Furthermore, nonindependence of nodes induced by processes acting across network connections (including social influence and homophily) is not the same as confounding that arises from unmeasured traits of nodes. The relationships that induce nonindependence are not features of the nodes themselves, but rather reflect relational connections linking nodes. In the case of social influence, the outcome of interest then *influences itself* based on the relationship structure amongst the nodes. Unmeasured confounding, in contrast, occurs when an observed association of variable Y with variable X in fact is due to by both Y and X being caused by a third unmeasured variable. Unmeasured confounding is well-known to cause biased estimates for slope coefficients in linear regression leading to statistical inconsistency, meaning that as more data are added to a regression, the model becomes more certain about a spurious association between measured variables when this association is, in fact, driven by the unmeasured variable(s) (Rohlf 2006; Lee 2021; Dow 1984; Naorli 1961).

**Fig. 1:**
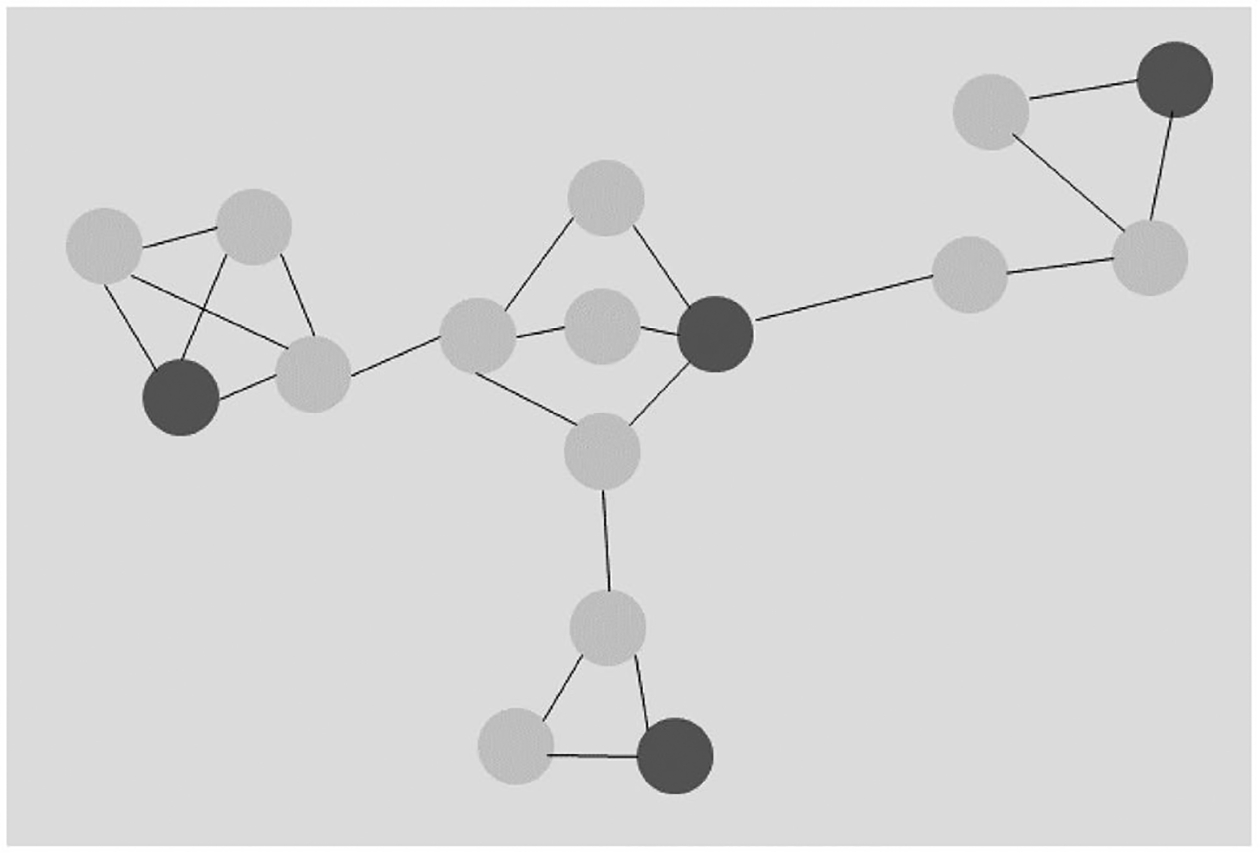
Illustration of how random sampling can select nodes that are relatively disconnected in a network and hence more statistically independent (relative to the complete network). Note: Dark gray nodes indicate those selected via random sampling

In the presence of network nonindependence, increased sample size does not yield biased slope estimates that are more consistent around an underlying value (unlike for confounding), leading some to the erroneous conclusion that nonindependence is therefore relatively unimportant. Rather, adding more complete data in the context of non-independence results in a different form of statistical inconsistency in which the model is more likely to find a significant result with a randomly positive or negative slope (Rohlf 2006). Somewhat paradoxically, reducing the sample size relative to the total population through sampling may in fact improve statistical performance for networked (nonindependent) data, because a randomly sampled subset may comprise nodes that are (generally) not directly linked together, and hence are statistically more independent (Fig. 1).

To our knowledge, the performance of random subsampling of network data has not been explored extensively in the social network analysis literature or in health policy research. It has been developed in the context of more hierarchical cross-cultural networks. Murdock and White’s seminal work creating the Standard Cross-Cultural Sample expressly used this approach to nonindependence to generate a dataset of relatively independent cultures that cross-cultural researchers could use for statistical analyses (Murdock and White 1969). The random sampling approach likely is ineffective specifically for highly hierarchical (tree-like) networks (Mace et al. 1994) but, hypothesizing that it may be effective for some social network contexts, we included it among others as a candidate method in our study.

Although the statistical problem of nonindependence in network data has been known for years, it never has been fully resolved. While there is an extensive methodological literature on the exponential random graph model (ERGM) that deals with the formation and dissolution of network connections in the context of homophily, there has been comparatively less methodological development to resolve pure cases of social influence. In the applied literature, studies employ a variety of methods—including the network autoregressive model, phylogenetic autoregression, robust standard errors, dyadic regression, and principal component analysis—that purport to solve the nonindependence problem (detailed below). Although the network autoregressive model is a primary approach used to address social influence, it is well-known to exhibit systematic biases regarding estimation of key parameters and has never been shown via simulation to produce acceptable statistical properties for Type I error (i.e., false findings of statistical significance) (Mizruchi and Neuman 2008; Neuman and Mizruchi 2010; Dittrich et al. 2017). To date, it is unclear which of these methods perform best, as their relative performance has not been directly evaluated in simulation studies.

To address this gap in the literature, we evaluate the relative performance of numerous analytic methods that have been used in the applied literature to correct for nonindependence arising from network social influence. We conducted a simulation study using simulated network data reflecting social influence processes occurring independently across multiple traits. While traits (and their diffusion) were simulated to be independent, the simulated social influence processes acting on traits means that nodes are not independent. Our simulation study compares various analytic methods for estimating the relationship between traits; methods that correct adequately for node nonindependence (either via a “robust” estimator or by transforming the network structure of the data) should infer no significant correlation between traits (estimating significant associations no more frequently than nominal significance level). To isolate the issue of nonindependence induced by network influence, our simulation assumed that the correct network structure was known with complete accuracy and that there was no confounding or homophily. If available statistical methods cannot correct for nonindependence under these conditions, they are very unlikely to perform well under real-world conditions in which these additional complexities like measurement inaccuracies, confounding, and homophily are common.

### 1.1 Statistical corrections for network nonindependence

We will focus on the context of estimating the association between outcome trait *Y* and predictor trait *X*. In simplest form, this could be assessed with a regression model of the form *y* = *Xβ* + *e*, in which the estimated coefficient $\hat{\beta }$ quantifies the association of interest. Interrelated network data violates the assumption of independence of observations (and in turn the *e* terms) underlying many regression methods such as ordinary least squares. Many statistical methods to account for nonindependence of network data have been proposed. Some methods attempt to correct the variance of regression coefficients (i.e., slopes or betas) for nonindependence, but they do so in different ways. Other methods correct the standard error of the slopes so as to obtain correct p-values for the slope (that may or may not be estimated correctly in terms of magnitude). Herein we briefly characterize each method that we tested, all of which have received full treatments in articles we cite. Table 1 shows a compact overview and comparison of the methods and is followed by brief descriptions of each.

*Network autoregression* models were developed specifically to deal with nonindependence in network data. They keep the relationship data in their natural dyadic form (i.e., connections from node A to B, A to C, etc.) and specify an autoregressive structure for the error term *e*. This approach divides the residual variation into a portion that is correlated across the network ties and then an uncorrelated residual as would be typical of any regression model (O’Malley 2008; Leenders 2002; Grafen 1989; Pagel 1999). We implemented this method using the *lnam* function in the “sna” R package (Butts 2020). Specifically, *lnam* fits the following linear network autocorrelation model: *y* = *Xβ* + *e*, with *e* = *σWe* + *υ*. In this model, *y* denotes a vector of the outcome trait, *X* is a covariate trait matrix, *W* is the adjacency matrix (with multiplicative factor *σ*), and the uncorrelated residual *υ* ~ *N*(0, *σ*^2^). *W* parameterizes the autocorrelation of each disturbance in *y* on its neighbors.

*Phylogenetic autoregression* applies the same general statistical approach as does network regression, but it first simplifies the network structure into a best-fitting bifurcating tree structure (Mathew and Perreault 2015a). The advantage of this approach is it greatly reduces the mathematical complexity and circularity of the original network. Prior simulation work has shown this method to be generally valid when the network is very close to a bifurcating tree structure (Matthews 2019), but this method presents a clear disadvantage if the network is not treelike and hence the phylogeny fails to represent aspects of the full network structure. We implemented this approach using the *hclust* function in R to perform hierarchical cluster analysis to identify the closest fitting tree for the network structure. We then used the *corPagel* function in the “ape” R package (Paradis and Schliep 2019) to estimate Pagel’s lambda correlation structure (*λ*) from a phylogenetic representation of the estimated tree. Again, the underlying model is *y* = *Xβ* + *e*, with *e* = *λWe* + *υ* but now *λ* is used as the scaling factor *σ* and *W* reflects the closest fitting tree. Finally, we regressed *y* on *X* using the the *gls* function in the R package “nlme” (Pinheiro et al. 2022), specifying the correlation structure as our estimated *λ*.

*Conley standard errors* is a method derived from spatial statistics that has been applied to deal with social influence on social networks (Schulz et al. 2019). Specifically, this method uses a Generalized Method of Moments approach and computes a variance–covariance matrix with spatial weights. While typically applied to geographic spatial data, this method can be applied in the context of social network data using spatial weights that represent the network distance between nodes as physical distances (e.g., pretending as if the social network space existed in a physical geographic space). In order to implement this method, one must transform the social network data into a two-dimensional array (e.g., representing latitude and longitude of nodes); we used principal component analysis to estimate 2 components from each network using the *isoMDS* function in the “MASS” R package (Venables and Ripley 2002). We then used the “conleyreg” R package (Düben et al. 2022) to estimating a regression model of the form *y* = *Xβ* + *e*, specifying the 2 estimated components as the “latitude” and “longitude” used to calculate the spatial weights.

*Random effects for network communities* model also adjusts the residual variation with a simplification, but it does this by first breaking up the network into a set of network communities. Membership in a network community then is assigned as a nominal variable to each individual, and this variable is entered into the model as a random effect; in other words, this approach fits a hierarchical linear model with network community as the grouping variable (Landon et al. 2018). In the literature this model also is known as a “clustered error” model or “mixed hierarchical” model. We inferred network communities with the *cluster walktrap* function in the “igraph” R package (Csardi and Nepusz 2005) and then used the *lmer* function in the “lme4” R package (Bates et al. 2015) to estimate a regression model of the form *y* = *Xβ* + *u* + *e*, where *u* denotes the network community random effect. Once again, while this simplifies the mathematical work needed to fit the regression, it may fail to account for important structural features of any given social network.

*Principal components from networks* first passes the network structure through a dimension reduction procedure to extract out several continuously varying variables. These are intended to recapitulate individuals’ positions in the network through a set of variables, which then are entered into the regression model as fixed effects (Mathew and Perreault 2015b). We used principal component analysis to estimate 5 components from each network using the *isoMDS* function in the “MASS” R package (Venables and Ripley 2002) and then estimated a linear regression model of the following form: *y* = *Xβ* + *β*_1_*C*_1_ + *β*_2_*C*_2_ + *β*_3_*C*_3_ + *β*_4_*C*_4_ + *β*_5_*C*_5_ + *e*, in which *C*_1_, …, *C*_5_ denote the 5 components.

*Robust standard errors* are an approach to estimate unbiased standard errors of OLS coefficients under certain violations the standard OLS assumption of independent and identically distributed (i.i.d.) error terms. In particular, robust standard errors can address heteroscedasticity (i.e., heterogenous variance of the error terms). Notably, robust standard errors do not require specifying the functional form of the underlying covariance matrix, unlike a weighted least squares approach. Although robust standard errors (and weighted least squares) were only ever designed to correct for the identically distributed assumption of i.i.d. (and not the independence assumption), we included it as a candidate method because robust standard errors have seen wide application in the literature far beyond their original intent (King and Roberts 2015). We implemented robust standard error estimation in R with the “sandwich” (Zeileis et al. 2020) and “lmtest” packages (Zeileis and Hothorn 2002), using the *coeftest* command (and specifying the “HC1” option) when estimating a regression model of the form *y* = *Xβ* + *e*.

*Dyadic regression with network covariates* is a technique we developed as part of this research that is based on prior network studies (Nooy 2011; O’Malley and Christakis 2011; Matthews et al. 2013). In this approach we first convert the traits into pairwise distances. We then regress these distance values, one for each pair of data points *y*_*dist*_ and *X*_*dist*_, against one another, while including a binary indicator term for the presence of a network tie between a pair (denoted *Z*). We also include two random effect terms corresponding to the two nodes comprising a given dyad. We fitted this model of the form *y*_*dist*_ = (*X*_*dist*_)*β* + *β*_1_*Z* + *u*_1_ + *u*_2_ + *e* with the *lmer* function in the lme4 package, where *u*_1_ and *u*_2_ denote node random effects.

*Random subsampling of network nodes* relies on the property that as fewer nodes are sampled from a complete network it becomes less likely the sampled nodes are connected. If all the sampled nodes are sufficiently distant from each other, i.e., separated by at least one intermediary node, then they can be treated as independent data points in statistical models. Even in large samples of the U.S. population like the General Social Survey, node independence can be assumed because the sample is far less than even 1% of the total population. Physician networks, however, have often been analyzed for nearly complete sets of physicians in entire markets (Brunson et al. 2018). Random subsampling may be viable in very large markets, but in smaller markets it may be intractable due to the obvious loss of statistical power this method entails. We implemented random subsampling by first selecting either a 10%, 30%, or 50% random sample of nodes and then estimating a standard ordinary least squares regression model of the form *y* = *Xβ* + *e*.

It is worth noting at this point that Matthews (2019) tested many of these same statistical corrections via simulation for highly hierarchical network structures derived from language trees (Matthews et al. 2019). That study found that random effects for network communities, principal components from networks, and phylogenetic autoregression all performed at acceptable levels for false positive rates when traits were simulated to diffuse via social influence across network ties that were tree-like. However, when the traits were inherited longitudinally down the branches of the tree, and without horizontal diffusion, only the autoregression method performed acceptably. In both forms of trait simulation, the network autoregression method had false positive rates at least 3 times greater than what they should have been. Thus, we acknowledge that network structural characteristics can matter a great deal to the performance of statistical models to deal with network data. In this current paper we are seeking to examine the types of social networks most commonly encountered in health policy research. For hierarchical (tree-like) networks, readers are recommended to consult Matthews (2019). Further research is needed to identify the effects of other types of network structures on model performance.

## 2 Methods

Our simulation analyses considered 2 data structure conditions: (1) fully simulated data with either 50 or 100 nodes and (2) partially simulated data, in which the network structure was empirically defined based on physician network with 914 nodes and node traits were simulated to diffuse via social influence. For both conditions, we simulated 500 datasets comprising 1,000 traits that each diffused independently on the network structures. That is, traits were neither correlated nor functionally related in our data. Generation of the social influence diffusion process for traits across networks was conducted as follows. For each trait, each node was initially assigned a trait value based on a random draw from a Gaussian distribution. For each node and each trait, the mean trait value among the node’s direct network connections was calculated and the node’s initial trait value was updated to by adding half of the distance between the initial value and the average value of their network. Traits were generated to be independent in order to assess the performance of methods with regard to Type I errors—in this setting, optimally-performing statistical methods should identify statistically significant correlations among the traits at or below the specified significance threshold, which we set at 0.05.

For the fully synthetic dataset, a Barabasi game was used to generate the simulated network structure using linear preferential attachment (the default for the *barabasi.game* function in the “igraph” R package). We used the Barabasi game as a starting point for our analysis because it is a well-known model in network science that has been used before in many simulation studies. The Barabasi game simulates the pattern common to real networks that nodes with many connections tend to acquire even more (left-skew degree distribution). We recognize, however, that it fails to approximate some features of real-world networks. For this reason, we also assessed performance on an empirical network comprising the largest network community among physicians who treat serious mental illness in the state of Colorado. We extracted this community with the *walktrap* algorithm on a network in which ties between physicians were inferred when they shared more than two patients in common using the “igraph” R package, Csardi and Nepusz (Csardi and Nepusz 2005). We interpret results that are common to both the simulated Barabasi networks and the empirical network to indicate robustness especially due to their many structural differences.

Having generated simulated diffused traits on networks (both simulated and empirical), we then assessed the relative performance of different analytic approaches. Beginning with naïve linear regression, we regressed one diffused trait as the outcome (*y*) of another randomly diffused trait (*X*) using a model of the form *y* = *Xβ* + *e* without any correction for nonindependence. The estimated slope coefficient for the predictor trait is the estimate of interest, and should be 0, as traits were generated to be independent. We also implemented 10 different statistical correction approaches described previously: robust standard errors; Conley standard errors; principal components; random effects; network autoregression; phylogenetic autoregression; dyadic regression; 10% random subsample; 30% random subsample; and 50% random subsample. For each method, we assessed 3 metrics of performance across the 500 simulation replications: bias of the estimated slope (average difference between the estimated $\hat{\beta }$ slope and true value of 0), estimated standard error, and the Type I error (frequency of incorrectly rejecting the null hypothesis, i.e., there truly is no effect). For techniques that demonstrated acceptable false positive rates, we then conducted simulations to estimate statistical power. For these simulations, traits were generated to be correlated, with correlation values between the traits ranging from 0.2 to 0.6 at intervals of 0.1 (generated using the function *corgen* in R package “ecodist” (Goslee and Urban 2007)). All analyses were conducted in using R version 4.0.4.

## 3 Results

**Fig. 2:**
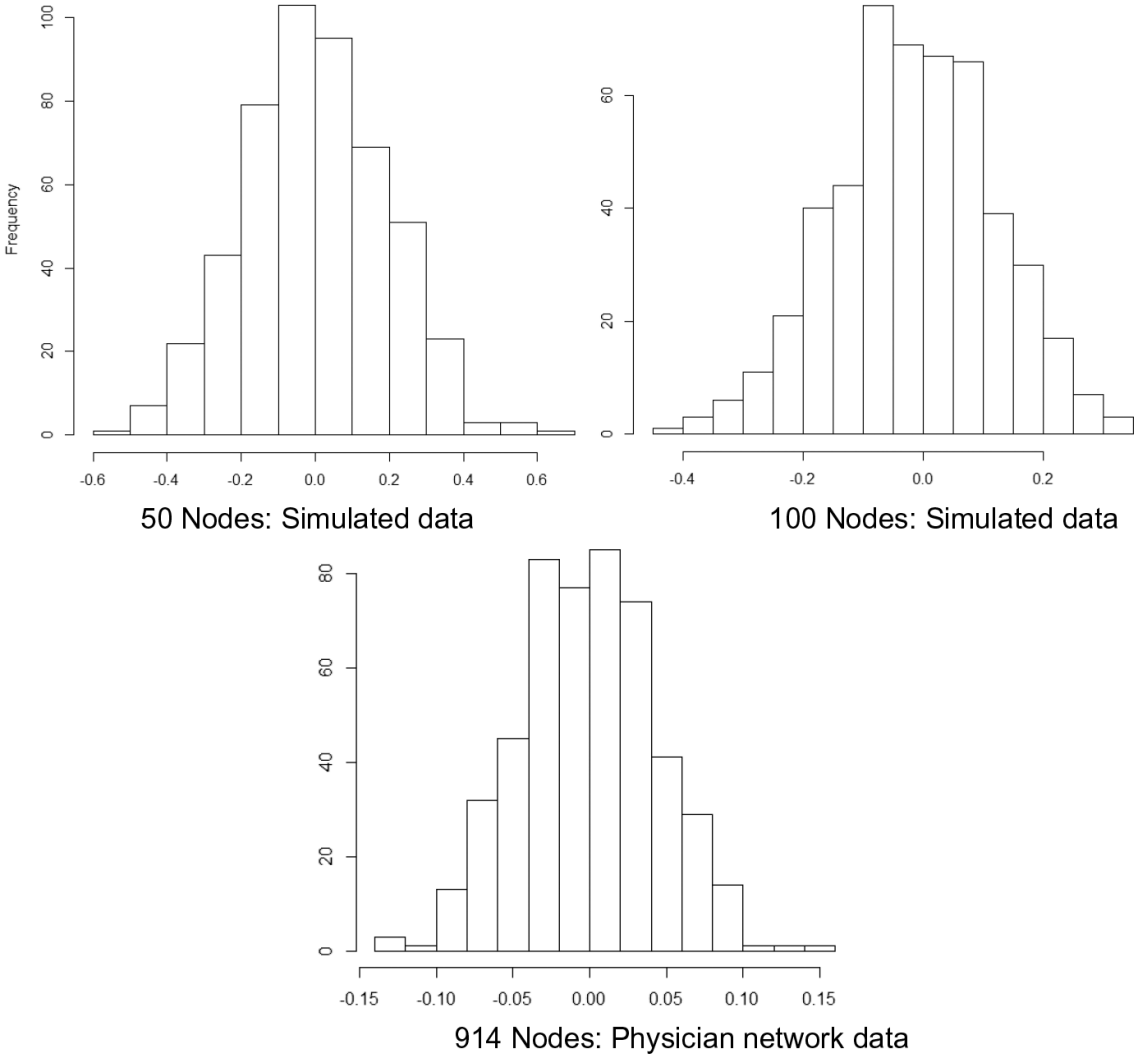
Histogram of slopes from linear regression of simulated traits on 50 and 100 node simulated networks, and the physician network

The statistical methods we tested all involved regressing an outcome (i.e., a given trait) against a hypothesized predictor (i.e., a different trait). In our simulations, in order to assess false positive rates, data were generated such that node traits were fully independent (had no relationship to each other). However, in all simulation conditions, traits were simulated to diffuse across the social network such that trait values of a node’s connections affected its own trait values, thereby reflecting a social influence process. This influence process results in non-independence of traits across nodes. Social influence is distinct from unmeasured confounding, in that nonindependence does not bias slope estimates while unmeasured confounding does. Indeed, histograms of slope estimates (Fig. 2) demonstrate that slope estimates were generally centered around zero, indicating minimal bias as expected.

Even though the slopes were centered on zero, the results from the linear regression models indicated that our simulated trait diffusion process reliably created datapoint non-independence and elevated the false positive rate (for randomly positive or negative slopes) (Table 2). Surprisingly, the two techniques that have received the most attention in the network science literature (random effects for network communities and network autoregression) did not adequately correct the elevated false positive rate. Methods adapted from the evolutionary science community (principal components and phylogenetic autoregression) did not correct the nonindependence problem either (Table 2). Methods derived from econometrics and spatial statistics, including robust standard errors and Conley standard errors, also failed to produce nominal type 1 error rates. Treating the simulated traits as distances in a dyadic regression, an adaptation of prior studies on relational data [De Nooy 2011; O’Malley 2011), also failed to lower false positive rate to the expected 0.05 (Table 2). Among the methods we tested, only randomly sampling to 10% of the original data successfully produced correct false positive rates.

It is notable, however, that among the methods that sought to use all the data by transforming residuals (robust standard errors, Conley standard errors, random effects for network communities, network autoregression, phylogenetic autoregression), the network autoregression method performed the best. This method was developed by network scientists for the purpose of correcting network nonindependence. Although it does not fully succeed in this goal, it shows the most promise at success, likely because it does not simplify the network structure, hence losing important statistical information, as do all the other methods that seek to correct nonindependence by transforming residual variation.

We estimated the statistical power for random sampling of 10% of the network, as this was the only method that exhibited acceptable proportions for false positive rate in the simulated networks and close to acceptable false positive rate proportions in the empirical physician network. The findings indicate that power was low when the networks had only 50 or 100 nodes. In the physician network of 914 nodes, statistical power to detect a significant result when traits were correlated was 85% or higher when the simulated correlations were 0.3 or greater (Table 3). In real world applications, power would likely be reduced from factors including inaccurate measurement error and inclusion of spurious control variables that are unrelated to the outcome. Variables are spurious when they are not actually related to the outcome variable but are included in a multiple regression as a putative control variable; this is a form of model misspecification that biases slope estimates toward zero and thereby reduces statistical power. While noting these limitations, our findings clearly demonstrate that, at least in principle, random sampling can achieve high statistical power while simultaneously yielding an appropriate Type I error rate (Table 3).

## 4 Discussion

Our simulations indicate that numerous analytic methods commonly used in applied network analyses do not adequately control for the statistical nonindependence that is introduced by processes in which nodes in a network (e.g., healthcare providers) influence one another. This calls into question research that has used standard regression-based methods to examine the relationships among variables determined in part by networks (Landon 2018; Barnett 2012; Pollack 2012). Such variables can include those measured from the network itself, such as number of connections or centrality, and also traits that are known to spread across network ties, such as physician prescribing practices.

What matters for statistical nonindependence to alter the false positive rate is not whether network measurements were part of a research design, but rather whether social influence in the network truly affects the traits of interest. If network influence is present, the data points are relationally connected within a network. In this context, conducting regression analyses of traits without any correction for network dynamics corresponds to the simple linear regression model in our simulations. Our results indicated that this approach yielded false positive rates that were three to four times larger than expected. Additionally, we observed that the common application of robust standard errors does not appropriately correct for nonindependence. This is consistent with recent literature that argues robust SEs are being used to correct for statistical violations well outside the context of their original development for heteroskedastic residuals, but that they are unlikely to actually fix the wide range of statistical problems to which empirical researchers are applying them (King and Roberts 2015).

A limitation of our current study is that we did not study how the statistical models perform under conditions of homophily or conditions of mixed homophily and influence. Although homophily can create identical patterns as does influence of trait correlations across network connections, it is unclear if the statistical models we tested would perform similarly under pure homophily conditions because homophily processes do not actually alter trait values of individuals based on network connections, whereas social influence definitely does this. Thus, the performance of the statistical models under homophily or mixed homophily/social influence conditions is an important matter for further research.

The many studies of practice patterns by physicians within a single hospital system provide concrete examples for the statistical nonindependence problem (Landon 2018; Barnett 2012; Pollack 2012). Physicians within a hospital system are likely linked together by a social network, and through this network likely influence each other about practice patterns and clinical norms. Studies that use regression in such a setting to compare practice patterns to other variables like cost and patient outcomes, but without accounting explicitly for network interdependence, may be subject to elevated false positive rates if included variables are affected by common network influence processes they share. Although traits like cost of patient care may at first glance seem to be wholly patient-driven rather than physician-driven features, the articles cited (Landon 2018; Barnett 2012; Pollack 2012) contended that physician medical judgement, in fact, plays an important role in cost. For example, with respect to judgements about the appropriate level of imaging needed for conditions ranging from kidney stones to back pain, prior work has shown that different physicians can make different judgements about patients with the same clinical presentation and physicians likely influence each other (via the physician network) about how to make these judgements. This type of process is how Pollack et al. (2012) explained higher cost of patient care for physician networks in which specialists are more central relative to costs of care in networks in which internal medicine physicians are more central (despite similar patient traits and outcomes across networks). The central physicians in these different networks may disproportionately influence their surrounding network physicians as to appropriate levels of diagnostics and procedures being applied to the same clinical presentations of patients, thereby influencing overall cost of care.

The results further suggest that research trends encouraging the use of complex “big data” may lead to erroneous findings if the data has an underlying network structure that is not accounted for by current analytic methods. For example, consider the increasingly common scenario in which a research group obtains the complete electronic health records from a major hospital system, then uses these data to evaluate the correlation of physician traits, such as the correlation between exposure to continuing medical education (CME) and a specific prescribing behavior targeted by CME. Under the likely scenario that physicians’ prescribing is influenced by discussions among themselves regarding prescribing, the study is at risk of conflating any effect of CME on prescribing with the effect on prescribing due to network influence, thereby potentially overestimating the impact of CME. Our results indicate that the deleterious effects of nonindependence would be reduced if such studies were to sample only a portion of the physicians in the system rather than use the complete physician population. Perhaps counterintuitively, our results suggest that in the presence of network structures, representative sampling will yield better statistical performance compared to complete data, but the latter often is perceived to be inherently better.

Unfortunately, existing procedures that incorporate complete network data into a regression model did not fully correct the problem or failed to correct it at all. In our simulations, the only method that generated accurate false positive rates was to randomly sample 10% of the nodes in network. In our applied example of physician network data, this method maintained false positive rates near nominal level and high power. Random sampling solves the statistical problem of nonindependence by restricting to nodes that are (generally) sufficiently distant from each other in the network, such that the assumption of statistical independence is plausible. Random sampling could be used to correct for network nonindependence even in cases in which the network structure has not been explicitly characterized. So long as the sampling is truly random with respect to the network structure, then our simulations suggest that sampling 10% or fewer nodes will separate them enough in the network to eliminate or largely mitigate the statistical effects of network non-independence. We note that in a specific empirical example, it likely would be judicious to perform the random sample over a number of iterations to ensure robusticity to sampling variance of any specific random 10%. We also note that, in practice, random sampling will be viable from a statistical power standpoint only when sample sizes are large (N > 1000) and/or when the true effects being estimated are large (Pearson *r* > 0.6).

It is intuitive to further suggest that systematically sampling nodes that are disconnected in the network might allow for more than 10% of the data to be retained, thereby increasing power while still eliminating nonindependence. We leave this as a question for further research, but we note that non-random sampling is likely a fraught effort because it very likely will not sample the attribute variables representatively. This non-representative sampling of attributes may be exacerbated by diffusion of those same attributes on the network, or by the attributes tending to induce network connections (homophily). Hence, although nonrandom sampling may conserve statistical power better than random sampling, it likely induces other statistical problems by reducing the representativeness of the sample. Overall, further statistical work is particularly needed to develop methods that correct for non-independence in network data while allowing use of the full data, thus preserving statistical power. Our simulations indicate that the network autoregression model shows the most promise for such further development.

## Supplementary Material

supplemental material 2

supplemental material 1

supplemental material 3

**Supplementary Information** The online version contains supplementary material available at https://doi.org/10.1007/s10742-023-00311-4.

## Figures and Tables

**Table 1:** Comparison of Methods to Correct for Nonindependence in Network Data

Method	Disciplinary Origin	Uses network data for correction?	Uses simplified approximation of network data?	Keeps network data dyadic or renders into variables?	Keeps attribute data as variables or renders into difference scores?
Network Autoregression	Social Network Analysis	Yes	No	Dyadic	Variables
Phylogenetic Autoregression	Evolutionary Biology/Anthropology	Yes	Yes	Dyadic	Variables
Conley Standard Errors	Spatial Statistics	Yes	Yes	Dyadic	Variables
Random Effects for Network Communities	Social Network Analysis	Yes	Yes	Variables	Variables
Principal Components from Networks	Anthropology	Yes	Yes	Variables	Variables
Robust Standard Errors	Statistics/Econometrics	No	NA	NA	Variables
Dyadic Regression with Network Covariates	Social Network Analysis	Yes	No	Dyadic	Difference Scores
Random Subsampling of Network Nodes	Anthropology	No	NA	NA	Variables

*NA* not applicable (i.e., method does not use network data for correction)

**Table 2:** Summary of statistical performance across candidate statistical methods

Statistical Correction Applied	Simulated Network 100 Nodes			Physician Network 914 Nodes		
	Observed Slope Bias	SE	Type I Error	Observed Slope Bias	SE	Type I Error
Simple Linear Reg	−0.015	0.100	15.6%	−0.003	0.033	13.8%
H-W Robust Standard Errors	−0.015	0.098	17.2%	−0.003	0.033	13.6%
Conley Standard Errors	−0.015	0.098	17.2%	−0.003	0.035	12.4%
Principal Components for Network	−0.013	0.103	11.2%	−0.004	0.033	13.0%
Random Effects for Network Communities	−0.013	0.099	16.2%	−0.002	0.033	12.0%
Network Autoregression	−0.009	0.098	10.8%	−0.001	0.033	8.6%
Phylogenetic Autoregression	−0.013	0.099	15.0%	−0.003	0.033	10.8%
Dyadic Regression	0.002	0.014	12.2%	0.000	0.002	10.2%
Subsample Random 10%	−0.024	0.354	4.4%	−0.005	0.106	6.6%
Subsample Random 30%	−0.006	0.188	7.0%	−0.003	0.060	7.8%
Subsample Random 50%	−0.008	0.142	10.2%	−0.005	0.047	9.8%

Each proportion estimate comes from 500 independent tests of significance among 1000 simulated traits. Simulations on a 50 node network produced very similar results (not shown). Observed Slope Bias close to zero indicates slope is unbiased across many iterations even though Type I Error is elevated (i.e., in a given iteration, the slope may be biased and misestimated)

**Table 3:** Power and slope estimates for random 10% sampling of network

Simulated correlation between variables	Simulation Type		
	Simulated Network	Simulated Network	Physician Network
	N = 50	N = 100	N = 914
	Statistical power (proportion significant results)		
0.2	4.6%	7.2%	47.6%
0.3	8.8%	12.0%	85.2%
0.4	9.4%	16.8%	98.0%
0.5	11.2%	32.0%	100.0%
0.6	19.2%	48.0%	100.0%

## References

[R1] Barnett ML, Landon BE, O’Malley AJ, Keating NL, Christakis NA: Mapping physician networks with self-reported and administrative data. Health Serv. Res 46, 1592–1609 (2011) 21521213 10.1111/j.1475-6773.2011.01262.x PMC3207194

[R2] Barnett ML, Christakis NA, O’Malley J, Onnela JP, Keating NL, Landon BE: Physician patient-sharing networks and the cost and intensity of care in US hospitals. Med. Care 50, 152–160 (2012) 22249922 10.1097/MLR.0b013e31822dcef7 PMC3260449

[R3] Bates D, Mächler M, Bolker B, Walker S: Fitting linear mixed-effects models using lme4. J. Stat. Softw 67, 1–48 (2015)

[R4] Berkman L, Glass T: Social integration, social methods, social support, and health. In: Berkman L, Kawachi I (eds.) Social Epidemiology, 137–173. New York, Oxford University Press (2000)

[R5] Breslau J, Dana B, Pincus H, Horvitz-Lennon M, Matthews L: Empirically identified networks of healthcare providers for adults with mental illness. BMC Health Serv. Res 21, (2021) 10.1186/s12913-021-06798-2 PMC8349008 34362369

[R6] Brunson JC, Laubenbacher RC: Applications of network analysis to routinely collected health care data: a systematic review. J. Am. Med. Inform. Assoc 25, 210–221 (2018) 29025116 10.1093/jamia/ocx052 PMC6664849

[R7] Butts CT 2020. sna: Tools for Social Network Analysis.

[R8] Csardi G, Nepusz T: The Igraph software package for complex network research. InterJ. Complex Syst (2005)

[R9] De Nooy W: Networks of action and events over time. A multilevel discrete-time event history model for longitudinal network data. Social Netw. 33, 31–40 (2011)

[R10] Dittrich D, Leenders RTAJ, Mulder J: Bayesian estimation of the network autocorrelation model. Social Netw. 48, 213–236 (2017)

[R11] Dow MM, Burton ML, White DR, Reitz KP: Galton’s problem as network autocorrelation. Am. Ethnol 11, 754–770 (1984)

[R12] Düben C, Bluhm R, Calderon L, Christensen D, Conley T, 2022. conleyreg: Estimations using conley standard errors

[R13] Felsenstein J: Phylogenies and the comparative method. Am. Nat 125, 1–15 (1985)

[R14] Gillespie A, Gardiner HM, Fink EL, Reese PP, Gadegbeku CA, Obradovic Z: Does sex, race, and the size of a kidney transplant candidate’s social network affect the number of living donor requests? A multicenter social network analysis of patients on the kidney transplant waitlist. Transplantation 104, 2632–2641 (2020) 33214495 10.1097/TP.0000000000003167 PMC8855970

[R15] Goslee SC, Urban DL: The ecodist package for dissimilarity-based analysis of ecological data. J. Stat. Softw 22, 1–19 (2007)

[R16] Grafen A: The phylogenetic regression. Philosoph. Trans. Royal Soc. London Ser.b Biol. Sci 326, 119–157 (1989) 10.1098/rstb.1989.0106 2575770

[R17] Grafen A, Hamilton WD: The phylogenetic regression. Philosoph. Trans. Royal Soc. London Ser. B Biol. Sci 326, 119–157 (1989) 10.1098/rstb.1989.0106 2575770

[R18] Hung M, Lauren E, Hon ES, Birmingham WC, Xu J, : Social network analysis of COVID-19 sentiments: application of artificial intelligence. J. Med. Internet Res 22, e22590 (2020) 32750001 10.2196/22590 PMC7438102

[R19] King G, Roberts ME: How robust standard errors expose methodological problems they do not fix, and what to do about it. Polit. Anal 23, 159–179 (2015)

[R20] Kuo YF, Agrawal P, Chou LN, Jupiter D, Raji MA: Assessing association between team structure and health outcome and cost by social network analysis. J. Am. Geriatr. Soc 69, 946 (2020) 10.1111/jgs.16962 PMC8166955 33289067

[R21] Landon BE, Keating NL, Onnela JP, Zaslavsky AM, Christakis NA, O’Malley AJ: Patient-sharing networks of physicians and health care utilization and spending among medicare beneficiaries. JAMA Intern. Med 178, 66–73 (2018) 29181504 10.1001/jamainternmed.2017.5034 PMC5833496

[R22] Lee Y, Ogburn EL: Network dependence can lead to spurious associations and invalid inference. J. Am. Stat. Assoc 116, 1060–1074 (2021)

[R23] Leenders R: Modeling social influence through network autocorrelation: constructing the weight matrix. Social Netw. 24, 21–47 (2002)

[R24] Mace R, Pagel M, Bowen JR, Gupta BKD, Otterbein KF, : The comparative method in anthropology [and comments and reply]. Curr. Anthropol 35, 549–564 (1994)

[R25] Manchanda P, Xie Y, Youn N: The role of targeted communication and contagion in product adoption. Mark. Sci 27, 961–976 (2008)

[R26] Mathew S, Perreault C Behavioural variation in 172 small-scale societies indicates that social learning is the main mode of human adaptation. Proc. Proc. R. Soc. B, 2015b, 282:2015b0061: The Royal Society 10.1098/rspb.2015.0061 PMC4590464 26085589

[R27] Mathew S, Perreault C: Behavioural variation in 172 small-scale societies indicates that social learning is the main mode of human adaptation. Proc. Royal Soc. b: Biol. Sci 282, 20150061 (2015a) 10.1098/rspb.2015.0061 PMC4590464 26085589

[R28] Matthews LJ: Dealing with culture as inherited information. In: Davis PK, O’Mahony A, Pfautz J (eds.) Social-Behavioral Modeling for Complex Systems, pp. 163–185. Wiley, Hoboken NJ (2019)

[R29] Matthews LJ, Edmonds J, Wildman WJ, Nunn CL: Cultural inheritance or cultural diffusion of religious violence? A quantitative case study of the Radical reformation. Relig. Brain Behav 3, 3–15 (2013)

[R30] Matthews L: Dealing with culture as inherited information. In: Davis PK, O’Mahony A, Pfautz J (eds.) Social-Behavioral Modeling for Complex Systems, 163–185. Hoboken, NJ, Wiley and Sons Inc (2019)

[R31] McCann M, Jordan JA, Higgins K, Moore L: Longitudinal social network analysis of peer, family, and school contextual influences on adolescent drinking frequency. J. Adolesc. Health 65, 350–358 (2019) 31196786 10.1016/j.jadohealth.2019.03.004 PMC6710020

[R32] Mizruchi MS, Neuman EJ: The effect of density on the level of bias in the network autocorrelation model. Social Netw. 30, 190–200 (2008)

[R33] Murdock GP, White DR: Standard cross-cultural sample. Ethnology 8, 329–369 (1969)

[R34] Naroll R: Two solutions to Galton’s problem. Philosophy of Science 28, 15–39 (1961a)

[R35] Naroll R: Two solutions to Galton’s problem. Philosophy of Science 28, 15–39 (1961b)

[R36] Neuman EJ, Mizruchi MS: Structure and bias in the network autocorrelation model. Social Netw. 32, 290–300 (2010)

[R37] O’Malley AJ, Marsden PV: The analysis of social networks. Health Serv. Outcomes Res. Method 8, 222–269 (2008) 10.1007/s10742-008-0041-z PMC2799303 20046802

[R38] O’Malley AJ, Christakis NA: Longitudinal analysis of large social networks: estimating the effect of health traits on changes in friendship ties. Stat. Med 30, 950–964 (2011) 21287589 10.1002/sim.4190 PMC3079434

[R39] Pagel M: Inferring the historical patterns of biological evolution. Nature 401, 877–884 (1999) 10553904 10.1038/44766

[R40] Paradis E, Schliep K: ape 5.0: an environment for modern phylogenetics and evolutionary analyses in R. Bioinformatics 35, 526–528 (2019) 30016406 10.1093/bioinformatics/bty633

[R41] Pinheiro J, Bates D, DebRoy S, Sarkar D, Heisterkamp S, 2022. nlme: Linear and nonlinear mixed effects models. R package version 3.1–157.

[R42] Pollack CE, Weissman G, Bekelman J, Liao K, Armstrong K: Physician social networks and variation in prostate cancer treatment in three cities. Health Serv. Res 47, 380–403 (2012a) 22092259 10.1111/j.1475-6773.2011.01331.x PMC3258347

[R43] Pollack CE, Weissman G, Bekelman J, Liao K, Armstrong K: Physician social networks and variation in prostate cancer treatment in three cities. Health Serv. Res 47, 380–403 (2012b) 22092259 10.1111/j.1475-6773.2011.01331.x PMC3258347

[R44] Rohlf FJ: A comment on phylogenetic correction. Evolution 60, 1509–1515 (2006) 16929667 10.1554/05-550.1

[R45] Schulz JF, Bahrami-Rad D, Beauchamp JP, Henrich J: The Church, intensive kinship, and global psychological variation. Science 366, eaau5141 (2019) 10.1126/science.aau5141 31699908

[R46] Shalizi CR, Thomas AC: Homophily and contagion are generically confounded in observational social network studies. Sociol. Methods Res 40, 211–239 (2011) 22523436 10.1177/0049124111404820 PMC3328971

[R47] Stein BD, Mendelsohn J, Gordon AJ, Dick AW, Burns RM, : Opioid analgesic and benzodiazepine prescribing among Medicaid-enrollees with opioid use disorders: the influence of provider communities. J. Addict. Dis 36, 14–22 (2017) 27449904 10.1080/10550887.2016.1211784 PMC5366980

[R48] Venables WN, Ripley BD: Modern applied statistics with S. Springer, New York (2002)

[R49] Zeileis A, Hothorn T: Diagnostic checking in regression relationships. R News 2, 7–10 (2002)

[R50] Zeileis A, Köll S, Graham N: Various versatile variances: an object-oriented implementation of clustered covariances in R. J. Stat. Softw 95, 1–36 (2020)

