# Supplementary material for: Evaluation via simulation of statistical corrections for network nonindependence
Source: Health Serv Outcomes Res Methodol. Author manuscript; Available in PMC 2025 Dec 14. (PMC12700765; doi:10.1007/s10742-023-00311-4)
Supplement: supplemental material 2 [file NIHMS2111777-supplement-supplemental_material_2.docx]

**Appendix 1b:** Simulation R code: Comparing performance across methods

##################################################################

library(igraph)

library(sandwich)

library(lmtest)

library(sna) ## linear autoregressive network approach

library(MASS) ## PCA

library(lme4) ## RE regression

library(ape) ## phylogenetic

library(nlme) ## phylogenetic

library(conleyreg) ## Conley SEs

##################################################################

**### load "diffsimcont.RData" in R and it will have the data needed for code below**

### select simulation set (choose one)

allsims<-diffsimcont50.2

# allsims<-diffsimcont100.2

### select the matching network stack (match on m)

simnet<-stack50.2

# simnet<-stack100.2

### select based on node number in networks

simnet.d<-array(dim=c(50,50,1000))

# simnet.d<-array(dim=c(100,100,1000))

simnet.norm<-simnet.d

# calculate shortest path

for (i in 1:500) {

temp.graph<-graph.edgelist(simnet[,,i],directed=F)

simnet.d[,,i]<-shortest.paths(temp.graph)

simnet.norm[,,i]<-(max(simnet.d[,,i])-simnet.d[,,i])/max(simnet.d[,,i])

simnet.norm[,,i][is.na(simnet.norm[,,i])]<-0

diag(simnet.norm[,,i])<-0

}

# calculate adjacency

simnet.adj<-simnet.d

for (i in 1:500) {

temp.graph<-simplify(graph.edgelist(simnet[,,i],directed=F))

simnet.adj[,,i]<-get.adjacency(temp.graph,sparse=F)

}

simnet.adj[1:5,1:5,1]

simnet.adj.d<-1-simnet.adj

node <- dim(allsims)[[1]]

##################################################################

### **METHOD 1: Simple regression: unadjusted + robust SE**

##################################################################

### Unadjusted SEs and robust SEs

lin.mod.results<-matrix(nrow=500,ncol=3)

robust1.mod.results<-matrix(nrow=500,ncol=3)

robust2.mod.results<-matrix(nrow=500,ncol=3)

robust3.mod.results<-matrix(nrow=500,ncol=3)

colnames(lin.mod.results)<-c("slope","SE", "p.value")

colnames(robust1.mod.results)<-c("slope","SE", "p.value")

colnames(robust2.mod.results)<-c("slope","SE", "p.value")

colnames(robust3.mod.results)<-c("slope","SE", "p.value")

for (i in 1:500) {

lin.mod<-lm(allsims[,,i]~allsims[,,(i+500)])

## Regular regression

lin.mod.results[i,]<-summary(lin.mod)$coefficients[2,c(1,2,4)]

## Robust SEs

rob1 <- coeftest(lin.mod, vcov=sandwich)

robust1.mod.results[i,]<-rob1[2,c(1,2,4)]

}

# add Type I error flag, calculate 95% CI, add coverage flag

type1_flag <- ifelse(lin.mod.results[,3]<0.05, 1, 0)

lb_temp <- lin.mod.results[,1] - qt(0.025,df=node-2,lower.tail=FALSE)*lin.mod.results[,2]

ub_temp <- lin.mod.results[,1] + qt(0.025,df=node-2,lower.tail=FALSE)*lin.mod.results[,2]

cov_flag <- ifelse(lb_temp>0 | ub_temp<0, 0, 1)

lin.mod.results <- cbind(lin.mod.results, cbind(type1_flag, lb_temp, ub_temp, cov_flag))

# add Type I error flag, calculate 95% CI, add coverage flag

type1_flag <- ifelse(robust1.mod.results[,3]<0.05, 1, 0)

lb_temp <- robust1.mod.results[,1] - qt(0.025,df=node-2,lower.tail=FALSE)*robust1.mod.results[,2]

ub_temp <- robust1.mod.results[,1] + qt(0.025,df=node-2,lower.tail=FALSE)*robust1.mod.results[,2]

cov_flag <- ifelse(lb_temp>0 | ub_temp<0, 0, 1)

robust1.mod.results <- cbind(robust1.mod.results, cbind(type1_flag, lb_temp, ub_temp, cov_flag))

##################################################################

### **METHOD 1a: Simple regression of RANDOM SAMPLE**

##################################################################

### 10% sample

samp10.lin.mod.results<-matrix(nrow=500,ncol=3)

colnames(samp10.lin.mod.results)<-c("slope","SE", "p.value")

for (i in 1:500) {

## select random sample

samp.size<-dim(allsims)[[1]]*0.1

rand.samp<-sample(1:dim(allsims)[[1]],samp.size,replace=F)

small.lin.mod<-lm(allsims[rand.samp,,i]~allsims[rand.samp,,(i+500)])

## Regular regression

samp10.lin.mod.results[i,]<-summary(small.lin.mod)$coefficients[2,c(1,2,4)]

}

# add Type I error flag, calculate 95% CI, add coverage flag

# adjust degrees of freedom for 10% sample size

type1_flag <- ifelse(samp10.lin.mod.results[,3]<0.05, 1, 0)

lb_temp <- samp10.lin.mod.results[,1] - qt(0.025,df=(0.1*node)-2,lower.tail=FALSE)*samp10.lin.mod.results[,2]

ub_temp <- samp10.lin.mod.results[,1] + qt(0.025,df=(0.1*node)-2,lower.tail=FALSE)*samp10.lin.mod.results[,2]

cov_flag <- ifelse(lb_temp>0 | ub_temp<0, 0, 1)

samp10.lin.mod.results <- cbind(samp10.lin.mod.results, cbind(type1_flag, lb_temp, ub_temp, cov_flag))

### 30% sample

samp30.lin.mod.results<-matrix(nrow=500,ncol=3)

colnames(samp30.lin.mod.results)<-c("slope","SE", "p.value")

for (i in 1:500) {

## select random sample

samp.size<-dim(allsims)[[1]]*0.3

rand.samp<-sample(1:dim(allsims)[[1]],samp.size,replace=F)

small.lin.mod<-lm(allsims[rand.samp,,i]~allsims[rand.samp,,(i+500)])

## Regular regression

samp30.lin.mod.results[i,]<-summary(small.lin.mod)$coefficients[2,c(1,2,4)]

}

# add Type I error flag, calculate 95% CI, add coverage flag

# adjust degrees of freedom for 30% sample size

type1_flag <- ifelse(samp30.lin.mod.results[,3]<0.05, 1, 0)

lb_temp <- samp30.lin.mod.results[,1] - qt(0.025,df=(0.3*node)-2,lower.tail=FALSE)*samp30.lin.mod.results[,2]

ub_temp <- samp30.lin.mod.results[,1] + qt(0.025,df=(0.3*node)-2,lower.tail=FALSE)*samp30.lin.mod.results[,2]

cov_flag <- ifelse(lb_temp>0 | ub_temp<0, 0, 1)

samp30.lin.mod.results <- cbind(samp30.lin.mod.results, cbind(type1_flag, lb_temp, ub_temp, cov_flag))

### 50% sample

samp50.lin.mod.results<-matrix(nrow=500,ncol=3)

colnames(samp50.lin.mod.results)<-c("slope","SE", "p.value")

for (i in 1:500) {

## select random sample

samp.size<-dim(allsims)[[1]]*0.5

rand.samp<-sample(1:dim(allsims)[[1]],samp.size,replace=F)

small.lin.mod<-lm(allsims[rand.samp,,i]~allsims[rand.samp,,(i+500)])

## Regular regression

samp50.lin.mod.results[i,]<-summary(small.lin.mod)$coefficients[2,c(1,2,4)]

}

# add Type I error flag, calculate 95% CI, add coverage flag

# adjust degrees of freedom for 50% sample size

type1_flag <- ifelse(samp50.lin.mod.results[,3]<0.05, 1, 0)

lb_temp <- samp50.lin.mod.results[,1] - qt(0.025,df=(0.5*node)-2,lower.tail=FALSE)*samp50.lin.mod.results[,2]

ub_temp <- samp50.lin.mod.results[,1] + qt(0.025,df=(0.5*node)-2,lower.tail=FALSE)*samp50.lin.mod.results[,2]

cov_flag <- ifelse(lb_temp>0 | ub_temp<0, 0, 1)

samp50.lin.mod.results <- cbind(samp50.lin.mod.results, cbind(type1_flag, lb_temp, ub_temp, cov_flag))

##################################################################

## **METHOD 2 - Linear Network Autoregressive Matrix**

##################################################################

lnam.mod.results<-matrix(nrow=500,ncol=3)

colnames(lnam.mod.results)<-c("slope","se", "p.value")

for(i in 1:500) {

### use adjacency matrix

lang.d.norm<-simnet.adj[,,i]

### Choose based on network size

xmat<-cbind(rep(1,50),allsims[,,(i+500)])

#xmat<-cbind(rep(1,100),allsims[,,(i+500)])

lnam.mod<-lnam(y=allsims[,,i],xmat,W2=lang.d.norm)

wald<-lnam.mod$beta[2]/lnam.mod$beta.se[2]

p.wald<-1-pchisq(wald^2,df=1)

lnam.mod.results[i,]<-c(lnam.mod$beta[2],lnam.mod$beta.se[2],p.wald)

print(i)

}

# add Type I error flag, calculate 95% CI, add coverage flag

type1_flag <- ifelse(lnam.mod.results[,3]<0.05, 1, 0)

lb_temp <- lnam.mod.results[,1] - qt(0.025, df=node-2,lower.tail=FALSE)*lnam.mod.results[,2]

ub_temp <- lnam.mod.results[,1] + qt(0.025, df=node-2,lower.tail=FALSE)*lnam.mod.results[,2]

cov_flag <- ifelse(lb_temp>0 | ub_temp<0, 0, 1)

lnam.mod.results <- cbind(lnam.mod.results, cbind(type1_flag, lb_temp, ub_temp, cov_flag))

##################################################################

### **METHOD 3: PRINCIPAL COMPONENTS**

##################################################################

pc.mod.results<-matrix(nrow=500,ncol=3)

colnames(pc.mod.results)<-c("slope","se", "p.value")

for (i in 1:500) {

## use shortest path (

lang.d<-simnet.d[,,i]

# non-dimensional scaling with 5 components (5 component representation of network)

reduce.lang.d<-isoMDS(as.matrix(lang.d),k=5)

# this regression adjusts for 5 components

pc.mod<-lm(allsims[,,i]~allsims[,,(i+500)] + reduce.lang.d$points[,1] + reduce.lang.d$points[,2] + reduce.lang.d$points[,3] + reduce.lang.d$points[,4] + reduce.lang.d$points[,5])

pc.mod.results[i,]<-summary(pc.mod)$coefficients[2,c(1,2,4)]

}

# add Type I error flag, calculate 95% CI, add coverage flag

# adjust degrees of freedom for inclusion of 5 components

type1_flag <- ifelse(pc.mod.results[,3]<0.05, 1, 0)

lb_temp <- pc.mod.results[,1] - qt(0.025, df = node-7,lower.tail = FALSE)*pc.mod.results[,2]

ub_temp <- pc.mod.results[,1] + qt(0.025, df = node-7,lower.tail = FALSE)*pc.mod.results[,2]

cov_flag <- ifelse(lb_temp>0 | ub_temp<0, 0, 1)

pc.mod.results <- cbind(pc.mod.results, cbind(type1_flag, lb_temp, ub_temp, cov_flag))

##################################################################

### **METHOD 4: RANDOM EFFECTS**

##################################################################

randeff.mod.results<-matrix(nrow=500,ncol=5)

colnames(randeff.mod.results)<-c("slope","se", "p.value", "ci_lb", "ci_ub")

for (i in 1:500) {

# obtains network communities

wt.comm<-cluster_walktrap(graph.adjacency(phys.adj,mode="undirected"))

# now includes RE with network community

randeff.mod<-lmer(allsims[,,i]~allsims[,,(i+500)]+(1|wt.comm$membership))

wald<-summary(randeff.mod)$coefficients[2,1]/ summary(randeff.mod)$coefficients[2,2]

p.wald<-1-pchisq(wald^2,df=1)

randeff.mod.results[i,]<-c(summary(randeff.mod)$coefficients[2,1], summary(randeff.mod)$coefficients[2,2],p.wald, confint(randeff.mod)[4,1], confint(randeff.mod)[4,2])

}

# add Type I error flag, add coverage flag

# obtaining CIs directly from RE model

type1_flag <- ifelse(randeff.mod.results[,3]<0.05, 1, 0)

cov_flag <- ifelse(randeff.mod.results[,4]>0 | randeff.mod.results[,5]<0, 0, 1)

randeff.mod.results <- cbind(randeff.mod.results, cbind(type1_flag, cov_flag))

##################################################################

### **METHOD 5: PHYLOGENETIC**

##################################################################

phylo.mod.results<-matrix(nrow=500,ncol=4)

colnames(phylo.mod.results)<-c("slope","se","p.value","matrix.tree.cor")

for (i in c(1:500)) {

# shortest path matrix

lang.d<-simnet.d[,,i]

lang.d.asdist<-as.dist(lang.d)

# find closest fitting tree

tree.d<-hclust(lang.d.asdist)

tree.d.distances<-cophenetic(tree.d)

# set up as phylo object

tree.phylo<-as.phylo(tree.d)

corlambda<-corPagel(value=1,phy=tree.phylo)

# format data

temp.data<-cbind(allsims[,,i],allsims[,,(i+500)])

colnames(temp.data)<-c("y","x")

temp.data<-as.data.frame(temp.data)

# Implement model

phylo.mod<-try(gls(y~x,correlation=corlambda,data=temp.data))

print(i)

# sometimes program hits error

if(class(phylo.mod)=="try-error") {

phylo.mod.results[i,1:3]<-c(NA,NA,NA)

} else {

# save estimate, SE, and p-value

phylo.mod.results[i,1:3]<-summary(phylo.mod)$tTable[2,c(1,2,4)]

# save matrix.tree.cor

phylo.mod.results[i,4]<-cor(tree.d.distances,lang.d.asdist)

}

}

phylo.mod.results<-na.omit(phylo.mod.results)

error <- rep(500-(dim(phylo.mod.results)[1]), dim(phylo.mod.results)[1])

# add Type I error flag, calculate 95% CI, add coverage flag

type1_flag <- ifelse(phylo.mod.results[,3]<0.05, 1, 0)

# calculate 95% CI and coverage rate

lb_temp <- phylo.mod.results[,1] - qt(0.025, df = node-2,lower.tail = FALSE)*phylo.mod.results[,2]

ub_temp <- phylo.mod.results[,1] + qt(0.025, df = node-2,lower.tail = FALSE)*phylo.mod.results[,2]

cov_flag <- ifelse(lb_temp>0 | ub_temp<0, 0, 1)

phylo.mod.results <- cbind(phylo.mod.results, cbind(type1_flag, lb_temp, ub_temp, cov_flag, error))

##################################################################

### **METHOD 6: DYADIC REGRESSION w NETWORK COVARIATES**

##################################################################

### This approach makes directed distances out of the variables

### and then uses random effects to control this distance-based regression.

mat.reg.results<-matrix(nrow=500,ncol=3)

colnames(mat.reg.results)<-c("slope","se", "p.value")

for (i in 1:500) {

temp.v1<-allsims[,,i]

temp.v2<-allsims[,,i+500]

# calculate row distance

tv1<-dist(temp.v1,method="manhattan")

tv2<-dist(temp.v2,method="manhattan")

mytemp <-cbind(tv1,tv2)

row.ids<-matrix(1:length(temp.v1),nrow=length(temp.v1),ncol=length(temp.v1), byrow=F)

col.ids<-matrix(1:length(temp.v1),nrow=length(temp.v1), ncol=length(temp.v1),byrow=T)

rid<-row.ids[lower.tri(row.ids)]

cid<-col.ids[lower.tri(col.ids)]

simnet.temp<-simnet.adj.d[,,i][lower.tri(simnet.adj.d[,,i])]

# regress distances on each other, including random effects

mat.reg<-lmer(mytemp[,1]~mytemp[,2]+simnet.temp+(1|rid)+(1|cid))

wald<-summary(mat.reg)$coefficients[2,1]/summary(mat.reg)$coefficients[2,2]

p.wald<-1-pchisq(wald^2,df=1)

mat.reg.results[i,]<-c(summary(mat.reg)$coefficients[2,1], summary(mat.reg)$coefficients[2,2],p.wald)

print(i)

}

# add Type I error flag, calculate 95% CI, add coverage flag

# obtaining CIs directly from RE model (stored in 4th and 5th column)

type1_flag <- ifelse(mat.reg.results[,3]<0.05, 1, 0)

#cov_flag <- ifelse(mat.reg.results[,4]>0 | mat.reg.results[,5]<0, 0, 1)

#mat.reg.results <- cbind(mat.reg.results, cbind(type1_flag, cov_flag))

mat.reg.results <- cbind(mat.reg.results, type1_flag)

##################################################################

### **METHOD 7: CONLEY STANDARD ERRORS**

### The input data to conleyreg must be spatial points in sf format

### or in a non-spatial data frame format (in long / lat format).

##################################################################

conley.mod.results<-matrix(nrow=500,ncol=3)

colnames(conley.mod.results)<-c("slope","se","p.value")

for (i in 1:500) {

#lang.d<-simnet.d[,,i]

lang.d<-simnet.d[,,i]

# run isoMDS with k=2 to provide lat/long input for conley

reduce.lang.d<-isoMDS(as.matrix(lang.d),k=2)

# format data for conley package: traits + lat/long

# must be data frame with var names

temp <- as.data.frame(allsims[,,1:1000])

temp <- cbind(temp,reduce.lang.d$points[,1],reduce.lang.d$points[,2])

names(temp)[1001]<-"lat"

names(temp)[1002]<-"long"

names(temp)[i]<-"y"

names(temp)[i+500]<-"x1"

# specify dist_cutoff in terms of km

con <- conleyreg(y ~ x1, data=temp, dist_cutoff=5, lat = "lat", lon = "long")

# save estimate, SE, and p-value

conley.mod.results[i,] <- con[2,c(1,2,4)]

}

# add Type I error flag, calculate 95% CI, add coverage flag

type1_flag <- ifelse(conley.mod.results[,3]<0.05, 1, 0)

lb_temp <- conley.mod.results[,1] - qt(0.025, df=node-2,lower.tail=FALSE)*conley.mod.results[,2]

ub_temp <- conley.mod.results[,1] + qt(0.025, df=node-2,lower.tail=FALSE)*conley.mod.results[,2]

cov_flag <- ifelse(lb_temp>0 | ub_temp<0, 0, 1)

conley.mod.results <- cbind(conley.mod.results, cbind(type1_flag, lb_temp, ub_temp, cov_flag))

##################################################################

## **COMPILE RESULTS**

##################################################################

final<-matrix(nrow=11,ncol=4)

colnames(final)<-c("bias","SE","TypeI","coverage")

## Bias stored in 1st column, SE stored in 2nd column

## Type I stored in 4th column, except RE models (6th column), or phylo (5th column)

## coverage stored in 7th column, except phylo (8th column)

SimpLin <- c(mean(lin.mod.results[,1]),mean(lin.mod.results[,2]), mean(lin.mod.results[,4]),mean(lin.mod.results[,7]))

RobustLin <- c(mean(robust1.mod.results[,1]),mean(robust1.mod.results[,2]), mean(robust1.mod.results[,4]),mean(robust1.mod.results[,7]))

Lin10 <- c(mean(samp10.lin.mod.results[,1]),mean(samp10.lin.mod.results[,2]), mean(samp10.lin.mod.results[,4]),mean(samp10.lin.mod.results[,7]))

Lin30 <- c(mean(samp30.lin.mod.results[,1]),mean(samp30.lin.mod.results[,2]), mean(samp30.lin.mod.results[,4]),mean(samp30.lin.mod.results[,7]))

Lin50 <- c(mean(samp50.lin.mod.results[,1]),mean(samp50.lin.mod.results[,2]), mean(samp50.lin.mod.results[,4]),mean(samp50.lin.mod.results[,7]))

Phylo <- c(mean(phylo.mod.results[,1]),mean(phylo.mod.results[,2]), mean(phylo.mod.results[,5]),mean(phylo.mod.results[,8]))

PrinComp <- c(mean(pc.mod.results[,1]),mean(pc.mod.results[,2]), mean(pc.mod.results[,4]),mean(pc.mod.results[,7]))

reLin <- c(mean(randeff.mod.results[,1]),mean(randeff.mod.results[,2]), mean(randeff.mod.results[,6]),mean(randeff.mod.results[,7]))

NetAR <- c(mean(lnam.mod.results[,1]),mean(lnam.mod.results[,2]), mean(lnam.mod.results[,4]),mean(lnam.mod.results[,7]))

#dyadic <- c(mean(mat.reg.results[,1]),mean(mat.reg.results[,2]), mean(mat.reg.results[,6]),mean(mat.reg.results[,7]))

Conley <- c(mean(conley.mod.results[,1]),mean(conley.mod.results[,2]), mean(conley.mod.results[,4]),mean(conley.mod.results[,7]))

final <- rbind(SimpLin,RobustLin,Conley,PrinComp,reLin,NetAR,Phylo,dyadic,Lin10,Lin30,Lin50)

final <- rbind(SimpLin,RobustLin,Conley,PrinComp,reLin,NetAR,Phylo,Lin10,Lin30,Lin50)

##################################################################

### POWER CALCULATIONS

##################################################################

library(ecodist)

## use corgen function to generate correlated data

## correlation: vary r = 0.20 to r = 0.60

corr.data <- matrix(nrow=node, ncol=1000)

for (i in 1:500) {

corr.data[,i] <- allsims[,,i]

corr.data[,i+500] <- corgen(x=corr.data[,i], r=0.2, epsilon=0.01)$y

}

## 10% sample

samp10.lin.mod.results.corr<-matrix(nrow=500,ncol=3)

colnames(samp10.lin.mod.results.corr)<-c("slope","SE", "p.value")

for (i in 1:500) {

## select random sample -- NOW USING CORRELATED DATA

samp.size<-dim(corr.data)[[1]]*0.1

rand.samp<-sample(1:dim(corr.data)[[1]], samp.size,replace=F)

## Regular regression

small.lin.mod.corr<-lm(corr.data[rand.samp,i] ~ corr.data[rand.samp,(i+500)])

samp10.lin.mod.results.corr[i,]<-summary(small.lin.mod.corr)$coefficients[2,c(1,2,4)]

}

# add power, add coverage flag

power <- ifelse(samp10.lin.mod.results.corr[,3]<0.05, 1, 0)

lb_temp <- samp10.lin.mod.results.corr[,1] - qt(0.025,df=(0.1*node)-2,lower.tail=FALSE)*samp10.lin.mod.results.corr[,2]

ub_temp <- samp10.lin.mod.results.corr[,1] + qt(0.025,df=(0.1*node)-2,lower.tail=FALSE)*samp10.lin.mod.results.corr[,2]

cov_flag <- ifelse(lb_temp>0 | ub_temp<0, 0, 1)

samp10.lin.mod.results.corr <- cbind(samp10.lin.mod.results.corr, cbind(power, lb_temp, ub_temp, cov_flag))

#calc power

mean(samp10.lin.mod.results.corr[,4])

##################################################################

##################################################################
