# Supplementary material for: Evaluation via simulation of statistical corrections for network nonindependence
Source: Health Serv Outcomes Res Methodol. Author manuscript; Available in PMC 2025 Dec 14. (PMC12700765; doi:10.1007/s10742-023-00311-4)
Supplement: supplemental material 1 [file NIHMS2111777-supplement-supplemental_material_1.docx]

**Appendix 1a:** Simulation R code: Generating network data for simulations

##################################################################

library(igraph)

library(stringr)

library(xlsx)

##################################################################

## SET RANDOM SEED

set.seed(5)

**## I. DEFINE NETWORK SIMULATION FUNCTION**

netsim <- function(x, y) {get.edgelist(barabasi.game(n = x, m = y, directed = FALSE))}

# Run simulations 1000 times each and store in arrays

stack20.2 <- replicate(500, netsim(20, 2))

stack20.5 <- replicate(500, netsim(20, 6))

stack20.10 <- replicate(500, netsim(20, 19))

**stack50.2 <- replicate(500, netsim(50, 3))**

stack50.5 <- replicate(500, netsim(50, 5))

stack50.10 <- replicate(500, netsim(50, 11))

**stack100.2 <- replicate(500, netsim(100, 2))**

stack100.5 <- replicate(500, netsim(100, 5))

stack100.10 <- replicate(500, netsim(100, 11))

stack1000.2 <- replicate(500, netsim(1000, 2))

stack1000.5 <- replicate(500, netsim(1000, 5))

stack1000.10 <- replicate(500, netsim(1000, 11))

**## II. CONTINUOUS CHARACTER DIFFUSION SIMULATION**

# 1. Define the continuous character network diffusion function

diffuse_cont <- function(network, influence, Ntaxa) {

net.rnorm <- network/rowSums(network)

value.t1 <- rnorm(n = Ntaxa)

ave.alter <- colSums(value.t1 * t(net.rnorm))

value.t2 <- value.t1 + (ave.alter - value.t1) * influence

allsims <- value.t2

return(allsims)

}

# 2. Run network diffusion function to create data of different dimensions

**## For network with n=50, m=2**

currentstack <- stack50.2

stacknum <- 50

# Create an array to hold simulation results.

diffsimcont50.2 <- array(dim = c(stacknum, 1, 1000))

# Start loop

for (i in 1:500) {

# Get adjacency matrices from the simulated networks

sim.g <- graph.edgelist(currentstack[,,i], directed = FALSE)

sim.adj <- get.adjacency(sim.g, sparse = FALSE)

# Store diffusion results

diffsimcont50.2[,,i] <- diffuse_cont(network = sim.adj, influence = 0.5, Ntaxa = stacknum)

diffsimcont50.2[,,i+500] <- diffuse_cont(network = sim.adj, influence = 0.5, Ntaxa = stacknum)

}

**## For network with n=100, m=2**

currentstack <- stack100.2

stacknum <- 100

# Create an array to hold simulation results.

diffsimcont100.2 <- array(dim = c(stacknum, 1, 1000))

# Start loop

for (i in 1:500) {

# Get adjacency matrices from the simulated networks

sim.g <- graph.edgelist(currentstack[,,i], directed = FALSE)

sim.adj <- get.adjacency(sim.g, sparse = FALSE)

# Store diffusion results

diffsimcont100.2[,,i] <- diffuse_cont(network = sim.adj, influence = 0.5, Ntaxa = stacknum)

diffsimcont100.2[,,i+500] <- diffuse_cont(network = sim.adj, influence = 0.5, Ntaxa = stacknum)

}

## CODE FOR ADDITIONAL NETWORK DIMENSIONS

## For network with n=20, m=2

currentstack <- stack20.2

stacknum <- 20

# Create an array to hold simulation results.

diffsimcont20.2 <- array(dim = c(stacknum, 1, 1000))

# Start loop

for (i in 1:500) {

# Get adjacency matrices from the simulated networks

sim.g <- graph.edgelist(currentstack[,,i], directed = FALSE)

sim.adj <- get.adjacency(sim.g, sparse = FALSE)

# Store diffusion results

diffsimcont20.2[,,i] <- diffuse_cont(network = sim.adj, influence = 0.5, Ntaxa = stacknum)

diffsimcont20.2[,,i+500] <- diffuse_cont(network = sim.adj, influence = 0.5, Ntaxa = stacknum)

}

## For network with n=20, m=5

currentstack <- stack20.5

stacknum <- 20

# Create an array to hold simulation results.

diffsimcont20.5 <- array(dim = c(stacknum, 1, 1000))

# Start loop

for (i in 1:500) {

# Get adjacency matrices from the simulated networks

sim.g <- graph.edgelist(currentstack[,,i], directed = FALSE)

sim.adj <- get.adjacency(sim.g, sparse = FALSE)

# Store diffusion results

diffsimcont20.5[,,i] <- diffuse_cont(network = sim.adj, influence = 0.5, Ntaxa = stacknum)

diffsimcont20.5[,,i+500] <- diffuse_cont(network = sim.adj, influence = 0.5, Ntaxa = stacknum)

}

## For network with n=20, m=10

currentstack <- stack20.10

stacknum <- 20

# Create an array to hold simulation results.

diffsimcont20.10 <- array(dim = c(stacknum, 1, 1000))

# Start loop

for (i in 1:500) {

# Get adjacency matrices from the simulated networks

sim.g <- graph.edgelist(currentstack[,,i], directed = FALSE)

sim.adj <- get.adjacency(sim.g, sparse = FALSE)

# Store diffusion results

diffsimcont20.10[,,i] <- diffuse_cont(network = sim.adj, influence = 0.5, Ntaxa = stacknum)

diffsimcont20.10[,,i+500] <- diffuse_cont(network = sim.adj, influence = 0.5, Ntaxa = stacknum)

}

## For network with n=50, m=5

currentstack <- stack50.5

stacknum <- 50

# Create an array to hold simulation results.

diffsimcont50.5 <- array(dim = c(stacknum, 1, 1000))

# Start loop

for (i in 1:500) {

# Get adjacency matrices from the simulated networks

sim.g <- graph.edgelist(currentstack[,,i], directed = FALSE)

sim.adj <- get.adjacency(sim.g, sparse = FALSE)

# Store diffusion results

diffsimcont50.5[,,i] <- diffuse_cont(network = sim.adj, influence = 0.5, Ntaxa = stacknum)

diffsimcont50.5[,,i+500] <- diffuse_cont(network = sim.adj, influence = 0.5, Ntaxa = stacknum)

}

## For network with n=50, m=10

currentstack <- stack50.10

stacknum <- 50

# Create an array to hold simulation results.

diffsimcont50.10 <- array(dim = c(stacknum, 1, 1000))

# Start loop

for (i in 1:500) {

# Get adjacency matrices from the simulated networks

sim.g <- graph.edgelist(currentstack[,,i], directed = FALSE)

sim.adj <- get.adjacency(sim.g, sparse = FALSE)

# Store diffusion results

diffsimcont50.10[,,i] <- diffuse_cont(network = sim.adj, influence = 0.5, Ntaxa = stacknum)

diffsimcont50.10[,,i+500] <- diffuse_cont(network = sim.adj, influence = 0.5, Ntaxa = stacknum)

}

## For network with n=100, m=5

currentstack <- stack100.5

stacknum <- 100

# Create an array to hold simulation results.

diffsimcont100.5 <- array(dim = c(stacknum, 1, 1000))

# Start loop

for (i in 1:500) {

# Get adjacency matrices from the simulated networks

sim.g <- graph.edgelist(currentstack[,,i], directed = FALSE)

sim.adj <- get.adjacency(sim.g, sparse = FALSE)

# Store diffusion results

diffsimcont100.5[,,i] <- diffuse_cont(network = sim.adj, influence = 0.5, Ntaxa = stacknum)

diffsimcont100.5[,,i+500] <- diffuse_cont(network = sim.adj, influence = 0.5, Ntaxa = stacknum)

}

## For network with n=100, m=10

currentstack <- stack100.10

stacknum <- 100

# Create an array to hold simulation results.

diffsimcont100.10 <- array(dim = c(stacknum, 1, 1000))

# Start loop

for (i in 1:500) {

# Get adjacency matrices from the simulated networks

sim.g <- graph.edgelist(currentstack[,,i], directed = FALSE)

sim.adj <- get.adjacency(sim.g, sparse = FALSE)

# Store diffusion results

diffsimcont100.10[,,i] <- diffuse_cont(network = sim.adj, influence = 0.5, Ntaxa = stacknum)

diffsimcont100.10[,,i+500] <- diffuse_cont(network = sim.adj, influence = 0.5, Ntaxa = stacknum)

}

## Create an array of generated data (m=2)

diffsimcont1000.2 <- array(dim = c(stacknum, 1, 1000))

# Start loop

for (i in 1:500) {

# Get adjacency matrices from the simulated networks

sim.g <- graph.edgelist(currentstack[,,i], directed = FALSE)

sim.adj <- get.adjacency(sim.g, sparse = FALSE)

# Store diffusion results

diffsimcont1000.2[,,i] <- diffuse_cont(network = sim.adj, influence = 0.5, Ntaxa = stacknum)

diffsimcont1000.2[,,i+500] <- diffuse_cont(network = sim.adj, influence = 0.5, Ntaxa = stacknum)

}

## Create an array of generated data (m=5)

diffsimcont1000.5 <- array(dim = c(stacknum, 1, 1000))

# Start loop

for (i in 1:500) {

# Get adjacency matrices from the simulated networks

sim.g <- graph.edgelist(currentstack[,,i], directed = FALSE)

sim.adj <- get.adjacency(sim.g, sparse = FALSE)

# Store diffusion results

diffsimcont1000.5[,,i] <- diffuse_cont(network = sim.adj, influence = 0.5, Ntaxa = stacknum)

diffsimcont1000.5[,,i+500] <- diffuse_cont(network = sim.adj, influence = 0.5, Ntaxa = stacknum)

}

## Create an array of generated data (m=10)

diffsimcont1000.10 <- array(dim = c(stacknum, 1, 1000))

# Start loop

for (i in 1:500) {

# Get adjacency matrices from the simulated networks

sim.g <- graph.edgelist(currentstack[,,i], directed = FALSE)

sim.adj <- get.adjacency(sim.g, sparse = FALSE)

# Store diffusion results

diffsimcont1000.10[,,i] <- diffuse_cont(network = sim.adj, influence = 0.5, Ntaxa = stacknum)

diffsimcont1000.10[,,i+500] <- diffuse_cont(network = sim.adj, influence = 0.5, Ntaxa = stacknum)

}

## Output results

save(list = ls(pattern = '^diffsimcont[1-9]+'), file = 'diffsimcont.RData')

##################################################################

##################################################################
